# Editorial: Abiotic and biotic stress responses of olive trees under climate change

**DOI:** 10.3389/fpls.2025.1677040

**Published:** 2025-09-01

**Authors:** Giovanni Caruso, Marco Romi, Giampiero Cai

**Affiliations:** ^1^ Department of Agriculture Food and Environment, University of Pisa, Pisa, Italy; ^2^ Department of Life Sciences, University of Siena, Siena, Italy

**Keywords:** climate resilience, abiotic stress, Xylella fastidiosa, genetic diversity, precision agriculture

The olive tree (*Olea europaea* L.), a cornerstone of agriculture in many arid regions, is facing increasing challenges due to climate change. Rising temperatures, decreasing rainfall and increased abiotic stresses such as drought and salinity, combined with the emergence of new pathogens, require innovative solutions to ensure sustainable cultivation. The Research Topic “Abiotic and Biotic Stress Responses of Olive Trees Under Climate Change” highlights a multidisciplinary approach to improve the resilience of this vital crop.

Resilience to abiotic stress is a central theme, with studies investigating the physiological and hydraulic responses of olive trees to drought and salinity. Imperiale et al. analyzed four olive cultivars - ‘Biancolilla’, ‘Calatina’, ‘Nocellara del Belice’ and ‘Koroneiki’ - and found significant differences in their hydraulic responses and biomass partitioning under water stress. For example, ‘Koroneiki’ maintained a high hydraulic conductance in its aerial parts, sustaining photosynthesis and growth, while ‘Calatina’ adopted a more conservative behavior, prioritizing root conductance and limiting transpiration. These cultivar-specific differences are crucial for selecting cultivars better suited to future climate scenarios.

Historical cultivar analysis supports this interpretation. Alkhatatbeh et al. studied cultivars such as ‘Nabali’, ‘Mehras’, ‘Frantoio’ and ‘Manzanillo’ under both drought and salinity stress. They observed a decrease in relative water content (RWC) and photosynthetic efficiency (Fv/Fm) in all varieties. ‘Nabali’ showed the highest salinity tolerance, while ‘Manzanillo’ and ‘Mehras’ had the most distinct differentially expressed genes (DEGs) under drought conditions. These DEGs represent stress-specific biomarkers that can be used in olive breeding and genetic improvement programs. Tadić et al. compared wild and cultivated olive genotypes and confirmed that drought had a more pronounced effect on shoot length, leaf area and dry mass compared to salinity. They also found that superoxide dismutase (SOD) activity was a reliable indicator of stress, in contrast to proline content. In addition to providing rich genetic diversity, wild genotypes remain a valuable resource for breeding programs.

At the molecular level, Narváez et al. clarified the role of the HPT and MPBQ MT genes in tocopherol (vitamin E) biosynthesis in the mesocarp of olive fruit. The expression of these genes is regulated by water status, temperature, light and wounding, suggesting their involvement in abiotic stress responses. Increased expression of HPT and MPBQ MT was found under regulated deficit irrigation (RDI) conditions, associated with higher tocopherol content, underscoring the role of HPT in drought stress resistance.

In addition to genetic selection, new strategies aim to improve the tolerance of olive trees. Gholami et al. demonstrated the effectiveness of foliar application of Fe2O3 graphitic carbon nanostructures (Fe2O3/g-C3N4) in mitigating the adverse effects of severe drought on ‘Shengeh’ cultivar. This treatment significantly improved pomological characteristics, oil content, photosynthetic efficiency, antioxidant enzyme activity, and uptake of essential ions such as Ca2+ and K+, while reducing Na+ accumulation. These results pave the way for the use of nanotechnology to improve resilience in agriculture.

At the same time, water management through precision agriculture techniques is increasingly important. Carella et al. combined proximal and remote sensors to assess the water status of the ‘Calatina’ cultivar. They established relationships between the thermal imagery derived crop water stress index (CWSI) and stem water potential (Ystem), as well as between different remote sensing vegetation indices (e.g. NDVI, WI, GNDVI) and crop water status. This study provides a basis for efficient irrigation systems that can optimize water use in water-scarce environments.

Finally, biotic threats, particularly the bacterium *Xylella fastidiosa*, represent an emerging stress factor exacerbated by climatic conditions. La Notte et al. conducted an extensive survey of natural olive resources in the Puglia epidemic area, identifying spontaneous genotypes resistant to *Xylella*. Parentage analysis revealed that the cultivar ‘Leccino’, known for its resistance, was a common parent for 67% of the progeny showing highly resistant, resistant or tolerant phenotypes. At the transcriptomic level, the highly resistant genotype S105 showed the least perturbation in gene expression in response to *Xylella* infection, indicating greater resilience compared to other resistant genotypes.

Resistance mechanisms include the ability to isolate the bacterium in xylem vessels and to cope with pathogen-induced water stress. This study confirms the value of ‘Leccino’ as a parent line for *Xylella* resistance breeding programs and provides gene targets for future genome editing techniques. Other pathogens, such as *Pseudomonas savastanoi*, which causes tumors on olive trees, are also being studied. Lavado-Benito et al. investigated the role of the GacA system in the virulence and competitiveness of this bacterium and demonstrated that GacA modulates motility, oxidative stress resistance, and virulence. Understanding these pathogen mechanisms is critical for developing integrated defense strategies that contribute to the overall health and resilience of olive trees.

In summary, the picture emerging from these studies is clear: olive production in the era of climate change requires an integrated approach. This includes identifying and exploiting genetic diversity (both existing cultivars and wild genotypes) to select more resilient cultivars, understanding the underlying molecular and physiological mechanisms of stress responses, applying advanced technologies for precision management, and developing novel interventions such as nanotechnology to enhance stress tolerance. These combined efforts are essential to ensure a sustainable future for the olive tree and its valuable products.

